# Cross-cultural validation and psychometric properties study of the Chinese college students’ life skills scale for sport—transfer scale

**DOI:** 10.3389/fpsyg.2024.1431239

**Published:** 2025-01-20

**Authors:** Lin Luo, Meilin He, Shaojing Wen, Junfeng Yuan, Huilin Xu

**Affiliations:** School of Physical Education, Guizhou Normal University, Guiyang, China

**Keywords:** cross-cultural validation, psychometric properties, sports transfer, life skills development, Chinese college students

## Abstract

**Introduction:**

This study aimed to conduct a cross-cultural validation of the Life Skills Scale for Sport—Transfer Scale (LSSS-TS) among Chinese college students and examine its psychometric properties.

**Methods:**

The study was conducted in three phases. In the first phase, four translation experts and five academic researchers collaborated to develop the Chinese version of the scale (C-LSSS-TS). Expert evaluations and feedback from 25 participants engaged in sports activities provided preliminary evidence for the scale’s construct validity. In the second phase, 409 participants were recruited, and factor analysis was performed to validate the structural dimensions of the scale. In the third phase, 168 participants completed both the C-LSSS-TS and a standard Life Skills Scale to examine the external construct validity.

**Results:**

The results demonstrated significant positive correlations between the subscales of sports transferable life skills and participants’ overall life skills development. These findings provided robust evidence for the structural validity of the C LSSS-TS.

**Conclusion:**

The C-LSSS-TS is a valid and practical tool for assessing life skills development among Chinese college sports participants. It offers researchers and practitioners an effective instrument to promote and evaluate life skills development in college students.

## Introduction

Over the past 15 years, sports activities have increasingly become a focal point of academic research as a significant avenue for promoting positive development among college students. This trend has been particularly highlighted in recent review articles and publications, especially those examining the role of sports in fostering the positive growth of college students (Holt et al., 2007; 2017). The Positive Youth Development (PYD) theory, which emphasizes building strengths and assets in young people, reframes youth as resources with potential rather than problems to be addressed (Holt, 2007), providing a robust theoretical foundation for such research. A key contribution of the PYD framework is its focus on the critical role of psychological well-being and life skills acquired through sports activities among college students (Aburn et al., 2016; Primo et al., 2023). Life skills, defined as the ability to effectively manage the demands and challenges of daily life (Pierce et al., 2017), have garnered significant attention in PYD literature facilitated by sports. Scholars have further emphasized the learnability, developmental nature, and adaptability of life skills (Danish et al., 2013), as well as their transferability to other domains, including academics, social relationships, and employment (Cronin, 2015).

The Life Skills Scale for Sport (LSSS), developed by Cronin and Allen (2017), serves as a tool for assessing eight core life skills acquired by adolescents through sports activities: teamwork, goal setting, social skills, problem-solving and decision-making abilities, emotional skills, leadership, time management, and interpersonal communication. This scale allows researchers to quantitatively evaluate the role of sports activities in life skills development and to investigate underlying theories and mechanisms, such as Self-Determination Theory (Ryan, 2017), that explain this process. Furthermore, it provides practitioners with a means to assess the effectiveness of sports-based life skills programs. However, the LSSS primarily focuses on the development of life skills within the sports context and does not fully address the transferability of these skills to other life domains.

While sports are widely recognized as an effective medium for acquiring life skills, the broader applicability of these skills—their relevance in addressing life challenges across various contexts—remains a critical area of inquiry. Developing specialized scales to measure skill transfer enables researchers and practitioners to better understand how participation in sports contributes to personal and societal development. The Life Skills Scale for Sport—Transfer Scale (LSSS-TS), introduced by Mossman et al. (2021), represents a significant advancement in this field. This scale is designed to assess the transferability of life skills from the sports environment to other domains, addressing gaps in existing research and providing a comprehensive tool for understanding the broader impact of sports on individuals. This approach underscores the importance of designing sports programs that not only facilitate skill acquisition but also promote the application of these skills in diverse life contexts.

However, the LSSS-TS currently exists only in English, limiting its applicability to non-native English-speaking populations. To address this limitation, developing a Chinese version of the LSSS-TS is essential for extending its use among Chinese college students. This study seeks to localize the LSSS-TS into Chinese through a series of systematic research efforts and to comprehensively evaluate its validity across multiple dimensions, including content, substantive, structural, and external constructs, in accordance with Messick’s (1995) unified validity framework. Additionally, the study aims to examine the universality of its construct validity to determine the scale’s suitability for evaluating life skills development among Chinese college students participating in sports activities.

## Study 1: content and substantive aspects of construct validity

Study 1 aimed to translate and adapt the Life Skills Scale for Sport—Transfer Scale (LSSS-TS), developed by Mossman et al. (2021), into Mandarin Chinese. This process focused on ensuring the relevance, representativeness, and technical quality of the scale items, which are core components of content and substantive validity (Messick, 1995). Content validity is defined as “the degree to which elements of an assessment instrument are relevant and representative of the constructs being measured” (Haynes et al., 1995, p. 239). Typically, content validity requires evaluation by recognized subject-matter experts to determine whether test items adequately measure well-defined constructs (Pasquali, 2009). The substantive aspect of construct validity emphasizes the importance of theories and process models in examining the domain processes involved in assessment tasks (Messick, 1995). According to Messick, this concept overlaps with content validity and includes appropriate sampling of domain processes, coverage of domain content, and evidence that respondents actively engage with the sampled processes.

The translation and adaptation procedure began with two bilingual experts with backgrounds in sports psychology and measurement translating the scale from English to Mandarin Chinese to ensure accuracy and professionalism. The initial translation draft was then reviewed by a panel of experts in sports science, psychology, and linguistics to evaluate the relevance, representativeness, and technical accuracy of the scale items during the language conversion process. Following this, two additional experts, who were not involved in the initial translation, conducted a back-translation to identify potential deviations or misunderstandings compared to the original English version. Necessary adjustments were made to address any discrepancies.

To address the substantive aspect of construct validity, the research team conducted a thorough analysis of the theoretical framework and process models underlying the scale to ensure that the translated version accurately reflected the domain processes involved in the assessment tasks. Additionally, the comprehensibility, relevance, and representativeness of the scale were evaluated through pilot testing with the target population. Feedback from the pilot study was used to refine and enhance the quality and adaptability of the scale. Finally, the revised version of the scale was submitted to a panel of domain experts for final review to ensure that the Chinese version faithfully captured the intent and measurement objectives of the original scale.

### Materials and methods

#### Participants

An expert panel was assembled for Study 1 to translate and cross-culturally adapt the Life Skills Scale for Sport—Transfer Scale (LSSS-TS) developed by Mossman et al. (2021). The panel consisted of nine members: four professional translators with extensive experience in translating scientific literature and academic backgrounds in English-speaking countries, and five academic researchers who were Chinese nationals, native Mandarin speakers, and held doctoral degrees in physical education or psychology with specializations in sports psychology. Additionally, a comprehension assessment study was conducted with 25 Chinese university students aged 18–29, representing the target population for the future application of the scale.

#### Instruments and procedures

The adapted instrument in this study was the LSSS-TS, originally developed by Mossman et al. (2021), comprising 40 measurement items. The scale evaluates life skills acquired by young people through sports activities, including teamwork (5 items), goal setting (5 items), social skills (5 items), problem-solving and decision-making (5 items), emotional skills (5 items), leadership (5 items), time management (5 items), and interpersonal communication (5 items). These eight skills are considered transferable to other life domains, such as social environments, family life, education, community involvement, and employment.

To ensure content and substantive validity, the LSSS-TS was first translated from English to Chinese. Using a 5-point Likert scale (1 = not at all, 5 = very much), expert panel members evaluated the theoretical relevance and linguistic clarity of each translated item. Specifically, they assessed clarity (e.g., ease of understanding) and relevance (e.g., whether the item should be included in the scale) and categorized the items into their respective subscales (e.g., assigning items to teamwork or leadership). Back-translation was then performed to verify the accuracy of the Chinese translation. During the translation and back-translation process, the expert panel discussed and resolved vocabulary discrepancies, making minor adjustments to item wording as needed.

A small-scale pilot study was conducted with 25 college students, who provided informed consent before completing the translated scale. Participants were asked to provide feedback on their understanding of the scale items. Based on their feedback, additional minor modifications were made to the wording of certain items, resulting in the finalized Chinese version of the LSSS-TS (C-LSSS-TS). The items were phrased in a way that reflected the application of life skills in broader contexts, such as “I apply teamwork skills learned from sports activities in social environments.”

#### Content validity data analysis

The content validity of the C-LSSS-TS, consisting of 40 items, was assessed to evaluate the consistency of expert judgments regarding the attribution of items to their designated dimensions (e.g., ensuring that teamwork-related items were correctly classified under the teamwork dimension). Content validity was analyzed by calculating the Content Validity Coefficient for Items (CCVi) for each item and the Content Validity Coefficient for the Scale (CCVt) for each dimension and the overall scale. A threshold of 0.80 was used to indicate sufficient content validity. To assess inter-rater reliability, Kappa coefficients were calculated, with values of 0.80 or higher considered acceptable.

### Results

The LSSS-TS was accurately translated and cross-culturally adapted into the C-LSSS-TS through the efforts of the expert translation and adaptation team, followed by piloting with university student sports participants. This process ensured the relevance, representativeness, and technical quality of the scale items, which are key components of content and substantive construct validity. The results of the content validity analysis further confirmed the linguistic clarity and practical relevance of the C-LSSS-TS items and their dimensions, with coefficients exceeding 0.813, indicating strong content validity (CCVi = 0.90 to 1.00; CCVt = 0.93–1.00). These findings suggest that the C-LSSS-TS provides clear and comprehensible linguistic expressions for Mandarin-speaking university student sports participants while maintaining close relevance to their sports backgrounds.

In the dimensions of teamwork, goal setting, social skills, problem-solving and decision-making, emotional skills, leadership, time management, and interpersonal communication, the consistency of item classification by experts, as measured by the Kappa coefficient, reached 0.853. This indicates that each item was accurately assigned to its intended latent dimension.

Overall, the findings from the expert panel and the university student sports participant sample provide robust evidence supporting the content and substantive construct validity of the C-LSSS-TS.

## Study 2: structural aspects of construct validity

The aim of Study 2 was to assess the structural validity of the C-LSSS-TS. Specifically, this study sought to validate the factor structure of scores obtained by a large sample of Chinese college student athletes and to test the internal consistency reliability of the subscale scores of the C-LSSS-TS.

### Methods and materials

#### Recruitment

Prior to data collection, ethical approval for the study was obtained from the Academic Ethics Committee of Guizhou Normal University. All participants provided informed consent before completing the questionnaire. The inclusion criteria required participants to have prior or current engagement in physical education and health courses at their school. Data collection was conducted between September and October 2023.

#### Measurement and participants

This study utilized the 40-item C-LSSS-TS scale, developed in Study 1, to assess participants’ perceptions of the application of eight life skills—teamwork, goal setting, time management, emotional skills, interpersonal communication, interpersonal skills, leadership, and problem-solving and decision-making—acquired through sports activities across five domains: social environments, family life, school education, community involvement, and career development. The full content of the C-LSSS-TS is presented in Supplementary material.

A total of 409 college students from various regions of China participated in the survey, completing the C-LSSS-TS questionnaire. The sample had a mean age of 20.33 years (SD = 8.98), a mean height of 167.02 cm (SD = 8.22), and a mean weight of 61.28 kg (SD = 13.87). Among the participants, 64.55% were male, and 52.57% identified as Han Chinese, with 12.96% coming from urban areas. By grade level, the sample consisted of 71.39% freshmen, 24.94% sophomores, 3.42% juniors, and 0.24% seniors. Regarding academic disciplines, 31.05% of students majored in humanities, 39.36% in natural sciences, 23.96% in engineering, and 5.62% in arts and sports.

The frequency distribution of participants’ weekly physical activity (excluding physical education classes) was also analyzed. The results showed that the majority of students (71.64%) participated in physical activities for 1 day per week. Additionally, 15.4% (63 students) engaged in physical activities for 2 to 3 days per week, while 12.96% (53 students) participated in such activities for 4 to 7 days per week. All participants were enrolled in school physical education courses.

#### Data analysis

To assess and validate the factor structure of the scores obtained from the C-LSSS-TS, this study employed Mplus software (Version 7.4; Kelloway, 2014) to conduct confirmatory factor analysis (CFA) using robust maximum likelihood estimation. This method was chosen due to the multivariate non-normality of the data, as indicated by Mardia’s (1970) multivariate kurtosis standardized estimate (77.98, *p* < 0.001), which far exceeded the threshold of 5.00 proposed by Bentler and Wu (2005). This result confirmed significant multivariate non-normality, consistent with recommendations in the literature (e.g., Chou et al., 1991).

Three models were tested in this study: (1) an eight-factor model representing all eight life skills acquired through sports and transferred to different domains; (2) a one-factor model representing overall sports transfer life skills; and (3) a bifactor CFA model, which included all eight transferable life skills factors as well as an overarching factor for overall sports transfer life skills. The bifactor CFA model was based on substantive measurement theory, as suggested by Mossman et al. (2021), whose research confirmed the presence of these two factors.

Model fit was evaluated using several fit indices: the ratio of the chi-square statistic to degrees of freedom (χ^2^/df), the root mean square error of approximation (RMSEA), the comparative fit index (CFI), and the Tucker-Lewis index (TLI). According to the criteria outlined by Tabachnick et al. (2013), a χ^2^/df value less than 3.0, RMSEA values below 0.08 (or 0.05 for excellent fit), and CFI and TLI values greater than 0.90 (or 0.95 for excellent fit) indicate good to excellent model fit.

To further evaluate the internal consistency reliability of the C-LSSS-TS subscales, Cronbach’s *α* coefficient and composite reliability (CR) were calculated based on factor loadings from the CFA results. A Cronbach’s α coefficient exceeding 0.70 was considered indicative of acceptable internal consistency reliability, while a composite reliability value greater than 0.70 was deemed appropriate, following the standards established by Kline (1999) and Tabachnick et al. (2013).

### Results

#### Factor structure evaluation

Table 1 presents the fit indices for the three CFA models. The results indicate that both the eight-factor model and the bifactor model demonstrated satisfactory fit, while the one-factor model exhibited poor fit. Notably, despite the potential negative impact of model size and complexity on fit indices (Cheung and Rensvold, 2002), the eight-factor model and the bifactor model—each comprising 40 items representing eight distinct transferable life skills—achieved satisfactory fit, providing encouraging evidence for the validity of the scale.

**Table 1:** C-LSSS-TS model fit indices.

Model	χ^2^	df	χ^2^/df	RMSEA	CFI	TLI
Eight-factor model	2136.079	712	3.000	0.070	0.900	0.917
First-order model	2840.165	740	3.838	0.084	0.785	0.824
Bifactor model	2202.394	722	3.050	0.073	0.907	0.912
Eight-factor model (male)	1791.872	712	2.517	0.076	0.903	0.893
Eight-factor model (female)	1850.771	712	2.599	0.087	0.878	0.892

Table 2 details the factor loadings for the eight-factor model and the bifactor model, both of which performed well in the fit assessment. For the eight-factor model, the average factor loading was 0.721, ranging from 0.593 to 0.816. In the bifactor model, all 40 items of the C-LSSS-TS significantly loaded on the general sports transfer life skills factor, with an average factor loading of 0.917 (ranging from 0.887 to 0.952). This finding suggests that the eight subscales of the C-LSSS-TS can be effectively integrated to compute an overall sports transfer life skills score. Additionally, the 40 items exhibited significant loadings on their respective specific sports transfer life skills factors, with an average factor loading of 0.717 (ranging from 0.598 to 0.815).

**Table 2:** Standardized factor loadings for the eight-factor and bifactor models.

Item	Standardized factor loadings	
	Eight-factor model	Bifactor model
TH1	0.593**	0.598**
TH2	0.662**	0.663**
TH3	0.604**	0.609**
TH4	0.740**	0.751**
TH5	0.767**	0.750**
GS1	0.687**	0.689**
GS2	0.735**	0.725**
GS3	0.659**	0.664**
GS4	0.726**	0.723**
GS5	0.714**	0.720**
IS1	0.728**	0.719**
IS2	0.708**	0.709**
IS3	0.699**	0.707**
IS4	0.722**	0.719**
IS5	0.713**	0.717**
PSD1	0.731**	0.724**
PSD2	0.743**	0.743**
PSD3	0.733**	0.737**
PSD4	0.793**	0.798**
PSD5	0.816**	0.815**
ES1	0.729**	0.731**
ES2	0.719**	0.718**
ES3	0.750**	0.752**
ES4	0.745**	0.739**
ES5	0.780**	0.782**
LD1	0.704**	0.700**
LD2	0.736**	0.737**
LD3	0.727**	0.736**
LD4	0.737**	0.738**
LD5	0.719**	0.714**
TM1	0.734**	0.729**
TM2	0.661**	0.664**
TM3	0.644**	0.638**
TM4	0.711**	0.704**
TM5	0.745**	0.757**
IC1	0.713**	0.710**
IC2	0.728**	0.730**
IC3	0.684**	0.685**
IC4	0.750**	0.755**
IC5	0.712**	0.709**

TH, Teamwork; GS, Goal setting; IS, Interpersonal skills; PSD, Problem solving and decision making; ES, Emotional skills; LD, Leadership; TM, Time management; IC, Interpersonal communication;** *p* < 0.01.

Even for items with standardized loading coefficients below 0.7—such as TH1, TH2, GS1, GS3, and IS3—significant loadings were observed on their respective specific sports transfer life skills factors. This indicates that these items align with the characteristics of their respective factors. Retaining these items is essential for maintaining the content and structural validity of the scale (Messick, 1995). Furthermore, the fit indices for the male, female, and mixed-gender models all fell within acceptable ranges, indicating that the model demonstrates good fit for both the overall sample and the subgroup samples.

Table 3 displays the correlation analysis among the eight sports transfer life skills. Correlation values ranged from 0.646 to 0.777, with an average of 0.708. Importantly, none of the correlation values exceeded 0.80, a threshold commonly used to identify inadequate discriminant validity between constructs (Brown and Schutte, 2006). This finding supports the discriminant validity of the scale and confirms the validity of its construct design.

**Table 3:** Correlations among the eight sport transferable life skills in study 2.

	1	2	3	4	5	6	7
1.Teamwork	–						
2.Goal setting	0.771**	–					
3.Interpersonal skills	0.742**	0.777**	–				
4.Problem solving and decision making	0.669**	0.739**	0.756**	–			
5.Emotional skill	0.692**	0.668**	0.718**	0.705**	–		
6.Leadership	0.659**	0.667**	0.710**	0.646	0.738**	–	
7.Time management	0.676**	0.677**	0.713**	0.683	0.735**	0.767**	–
8.Interpersonal communication	0.654**	0.652**	0.688**	0.711**	0.732**	0.693**	0.772**

**p < 0.01.

#### Internal consistency reliability

The internal consistency reliability of each subscale, as well as the overall C-LSSS-TS, was assessed using Cronbach’s alpha coefficients. The results were as follows: Teamwork (α = 0.804), Goal Setting (α = 0.830), Social Skills (α = 0.839), Problem Solving and Decision Making (α = 0.873), Emotional Skills (α = 0.860), Leadership Skills (α = 0.846), Time Management (α = 0.829), and Interpersonal Communication (α = 0.841). All coefficients exceeded the threshold of 0.70 recommended by Nunnally and Bernstein (1994), indicating high internal consistency reliability for each subscale.

The Cronbach’s alpha coefficient for the overall C-LSSS-TS was 0.969, substantially higher than the standard of 0.70 proposed by Tabachnick et al. (2013). This result further confirms the comprehensive reliability of the research instrument.

## Study 3: external aspect of construct validity

The aim of this study was to examine whether the subscale scores of the C-LSSS-TS (i.e., the eight Sport Transferable Life Skills subscales and the overall C-LSSS-TS score) were correlated with theoretically relevant outcomes, thereby testing the external aspect of construct validity (Messick, 1995). To assess external validity, we selected the Vocational College Students Life Skills Assessment Scale, developed by Chinese scholars, as the measurement tool.

Life skills refer to a range of abilities that enable individuals to effectively cope with daily life challenges and tasks. According to the World Health Organization (2009), these skills can be categorized into three aspects: critical thinking and decision-making skills, interpersonal relationships and communication skills, and coping and self-management skills. Sport transferable life skills, on the other hand, specifically refer to the application of these life skills—acquired through participation in sports—to other areas of daily life, such as education, family, and work (Mossman et al., 2021).

In essence, life skills represent a foundational set of abilities, while sport transferable life skills emphasize the transfer and application of these abilities from the sports domain to other life domains. This relationship highlights that sports activities not only promote physical health but also provide individuals with a platform to learn, develop, and apply key life skills in various life contexts (Goudeau and Baker, 2021).

### Methods and materials

#### Participants and measures

The sample for this study consisted of 168 university students, including 100 males and 68 females, aged between 18 and 19 years (mean age = 18.863 years, standard deviation = 0.345). In terms of ethnicity, 49.40% of the participants were Han Chinese, while 17.26% came from urban backgrounds. Regarding academic disciplines, 36.31% of the students majored in humanities, 36.31% in sciences, 23.21% in engineering, and 4.17% in arts and sports.

To assess participants’ development of sport transferable life skills, the 40-item C-LSSS-TS scale, as described in previous research, was employed. Based on the current sample (see Table 4), the Cronbach’s α coefficients for the subscales of the C-LSSS-TS ranged from 0.817 to 0.912, indicating high internal consistency reliability for the subscales (Nunnally and Bernstein, 1994).

**Table 4:** Correlation between participants’ C-LSSS-TS and life skills components.

	Cognition and thinking (0.976)	Self-management (0.796)	Interpersonal communication (0.935)	Life skills (0.977)
Teamwork (0.817)	0.355**	0.369**	0.405**	0.378**
Goal setting (0.869)	0.379**	0.378**	0.434**	0.405**
Interpersonal skills (0.842)	0.317**	0.333**	0.370**	0.342**
Problem solving and decision making (0.912)	0.331**	0.399**	0.392**	0.397**
Emotional skill (0.855)	0.321**	0.382**	0.403**	0.396**
Leadership (0.884)	0.317**	0.250**	0.276**	0.279**
Time management (0.867)	0.341**	0.324**	0.388**	0.361**
Interpersonal communication (0.854)	0.301**	0.328**	0.339**	0.329**
C-LSSS-TS (0.969)	0.385**	0.400**	0.435**	0.418**

*N* = 168. Correlation coefficients are Pearson’s product–moment correlation coefficients. Cronbach’s α coefficients for each subscale are presented in parentheses. **p < 0.01.

Participants’ life skills were further assessed using the Vocational College Students Life Skills Assessment Scale developed by Subasree et al. (2014). This scale consists of 100 items divided into three dimensions and 10 specific abilities. The first dimension, cognitive and thinking abilities, includes self-awareness, problem-solving, decision-making, creative thinking, and critical thinking, with 10 items for each ability. The second dimension, interpersonal communication abilities, encompasses effective communication, empathy, and interpersonal relationships, each assessed with 10 items. The third dimension, self-management abilities, covers emotional management and stress management, also with 10 items each. Participants rated their responses on a 5-point Likert scale (1 = strongly disagree to 5 = strongly agree), which included 95 positively worded items and 5 negatively worded items.

Previous research has demonstrated the validity and reliability of the Vocational College Students Life Skills Assessment Scale (Ouyang Yuliang et al., 2017). Consistent with the current sample, the subscales of this scale exhibited Cronbach’s *α* coefficients ranging from 0.796 to 0.976, further confirming its reliability (Nunnally and Bernstein, 1994).

#### Data analysis

Pearson product–moment correlation coefficients were used in this study to examine the relationships between perceived life skills development and various sport transferable life skills factors measured by the C-LSSS-TS. Statistical significance was set at *p* < 0.05. Following Cohen’s (1988) criteria, correlations were categorized as small (*r* = ±0.10 to ±0.29), medium (*r* = ±0.30 to ±0.49), and large (*r* > ±0.50).

### Results

The results presented in Table 4 reveal significant positive correlations between the eight specific sport transferable life skills and the overall sport transferable life skills with the subscales of the life skills scale (i.e., cognitive and thinking, self-management, and interpersonal communication). Correlation coefficients ranged from 0.276 to 0.435, indicating moderate positive associations between various abilities. These findings suggest that proficiency in one domain is positively associated with enhanced proficiency in other domains.

The substantial correlations between cognitive and thinking, self-management, and interpersonal communication skills with dimensions such as teamwork, goal setting, interpersonal skills, problem-solving and decision-making, emotional skills, leadership, and time management highlight the interconnected nature of these abilities. The cumulative scores of the C-LSSS-TS further validate the interdependence of these skills, emphasizing the importance of an integrated skill framework for personal and professional development in individuals with sport transferable life skills.

This analysis underscores the multifaceted role of sport transferable life skills in fostering overall development, demonstrating their capacity to enhance a wide range of life skills that contribute to success across various domains.

## Discussion

This study involved the translation and adaptation of the original LSSS-TS scale, which was initially designed for professional sports participants, into a version suitable for university students engaged in sports activities. This adaptation required shifting the target population from individuals with professional sports backgrounds to a broader cohort of university students, who differ significantly in their levels of sports participation, life skills requirements, and psychological and social contexts compared to the original scale’s intended audience.

During the translation and adaptation process, an expert panel was established, consisting of professional translators and academic experts in education and sports psychology. These experts, with extensive experience in translation and academic research in English-speaking countries, provided robust support for the translation effort (Hambleton et al., 2004; Ji et al., 2022). Challenges such as linguistic differences and cultural disparities were addressed through several measures. These included evaluating the theoretical relevance and linguistic clarity of translated items using a 5-point Likert scale, ensuring semantic consistency with the original text, and enhancing ease of comprehension for participants. Back-translation, vocabulary discussions within the expert panel, and adjustments to item wording were conducted to ensure translation accuracy and fluency (Sousa and Rojjanasrirat, 2011). Given the differences in the sample population, particular attention was paid to cultural adaptation and linguistic expression to ensure the scale’s applicability to a broader university student demographic. To further refine the scale, a small-scale comprehension assessment was conducted with 25 Chinese university students aged 18 to 29 who participated in sports activities. Feedback from this group led to additional modifications to the wording of scale items, improving both accuracy and clarity.

The structural validity of the C-LSSS-TS scale was confirmed through factor analysis. Confirmatory factor analysis (CFA) using Mplus software demonstrated a good fit for both the eight-factor model and the bifactor model, affirming the scale’s structural validity among Chinese university students (Kline, 2023). Each factor’s structure and interpretability were analyzed in detail, highlighting the life skills domains represented by each factor and their application across various contexts, including social, familial, educational, communal, and occupational settings (Brown, 2015).

The internal consistency of the C-LSSS-TS scale was also evaluated. Cronbach’s *α* coefficients and composite reliability values, calculated based on factor loadings, indicated high internal consistency and stability for the subscales and overall scores (Nunnally and Bernstein, 1994). These findings support the scale’s reliability and its effectiveness in assessing the transfer of life skills through sports.

The findings of this study underscore the positive effects of sports activities on the development of individual life skills and highlight the importance of integrating these insights into physical education and sports training programs. By fostering transferable life skills, sports can contribute to personal and professional development, enhancing students’ abilities to navigate various life domains (Weinberg and Gould, 2023).

These results also emphasize the need for continuous refinement of research designs and methods to deepen the understanding of the positive effects of sports on individuals. Such efforts can lead to the development of more effective strategies and interventions to promote health, personal growth, and education through sports (Hackney and Viru, 2008).

Future research should explore the scale’s applicability and validity among university students from diverse cultural and educational backgrounds. This would enhance the scale’s universality and provide more specific insights into educational practices, thereby supporting university students’ sports participation and life skills development more comprehensively. Expanding the research to include diverse populations will also help refine the scale further, ensuring its relevance and effectiveness in promoting life skills through sports across different contexts.

### Limitations and future research suggestions

Firstly, the research sample primarily consisted of university students from a specific region in China, which may introduce geographical and cultural constraints, limiting the generalizability of the findings. Additionally, in the sample used in Study 2, the proportion of males was significantly higher than that of females, potentially affecting the results due to the uneven gender distribution. Future studies should strive for a more balanced gender ratio in the sample. Secondly, the study relied on self-reported questionnaire data, which may be subject to self-reporting biases. Future research could address this limitation by incorporating objective evaluation measures for validation. Furthermore, although some potential confounding variables were controlled, other unexamined factors may still influence the results, warranting further investigation.

To address these limitations, future research could focus on the following areas: expanding the sample to include university students from diverse regions and demographic groups to test the generalizability and robustness of the scale; employing field observations or experimental methods to explore the mechanisms through which sports activities influence individual life skills; and refining the scale’s content and structure to improve its applicability and validity across different cultural contexts and populations. By continuously improving research designs and methodologies, a deeper understanding of the positive effects of sports activities on individuals can be achieved, providing more effective strategies and interventions to promote health, personal development, and education.

## Conclusion

The translation and adaptation process successfully transformed the original LSSS-TS scale into the C-LSSS-TS, which was validated for use among Chinese university students through analyzes of structural validity, internal consistency, and external validity. These findings have significant implications for promoting sports participation and fostering the development of life skills among university students. However, certain limitations of the study should be acknowledged. Future research is encouraged to expand the sample scope, incorporate objective evaluation indicators for validation, and further explore the mechanisms through which sports activities influence life skills development.

## Data Availability

The raw data supporting the conclusions of this article will be made available by the authors, without undue reservation.

## References

[ref1] Aburn G. Gott M. Hoare K. (2016). What is resilience? An integrative review of the empirical literature. J. Adv. Nurs. 72, 980–1000. doi: 10.1111/jan.12888 26748456

[ref3] Bentler P. M. Wu E. J. (2005). EQS 6.1 for windows. Structural equations program manual. Encino, CA: Multivariate Software.

[ref4] Brown T. A. (2015). Confirmatory factor analysis for applied research. New York: Guilford Publications.

[ref9001] Brown R. F. Schutte N. S. (2006). Direct and indirect relationships between emotional intelligence and subjective fatigue in university students. J. Psychosom. Res. 60, 585–593. doi: 10.1016/j.jpsychores.2006.05.001 16731233

[ref9002] Cheung G. W. Rensvold R. B. (2002). Evaluating goodness-of-fit indexes for testing measurement invariance. Struct. Equ. Model. 9, 233–255. doi: 10.1207/S15328007SEM0902_5

[ref5] Chou C. P. Bentler P. M. Satorra A. (1991). Scaled test statistics and robust standard errors for non-normal data in covariance structure analysis: a Monte Carlo study. Br. J. Math. Stat. Psychol. 44, 347–357. doi: 10.1111/j.2044-8317.1991.tb00966.x 1772802

[ref9003] Cohen S. (1988). Psychosocial models of the role of social support in the etiology of physical disease. Health Psychol. 7:269. 3289916 10.1037//0278-6133.7.3.269

[ref6] Cronin L. (2015). Life skills development through youth sport: Antecedents, consequences, and measurement. University of Stirling.

[ref7] Cronin L. D. Allen J. (2017). Development and initial validation of the life skills scale for sport. Psychol. Sport Exerc. 28, 105–119. doi: 10.1016/j.psychsport.2016.11.001

[ref8] Danish S. J. Forneris T. Wallace I. (2013). “Sport-based life skills programming in the schools” in School sport psychology (London: Routledge), 41–62.

[ref11] Goudeau S. M. Baker B. L. (2021). Developing effective youth-adult relationship: perspectives of adult volunteers in physical-activity based youth development programs. J. Nonprofit Publ. Sect. Market. 33, 539–563. doi: 10.1080/10495142.2020.1760998

[ref13] Hackney A. C. Viru A. (2008). Research methodology: endocrinologic measurements in exercise science and sports medicine. J. Athl. Train. 43, 631–639. doi: 10.4085/1062-6050-43.6.631 19030142 PMC2582556

[ref14] Hambleton R. K. Merenda P. F. Spielberger C. D. (2004). Adapting educational and psychological tests for cross-cultural assessment. London: Psychology Press.

[ref9004] Haynes S. N. Richard D. Kubany E. S. (1995). Content validity in psychological assessment: A functional approach to concepts and methods. Psychol. Assess. 7:238. doi: 10.1037/1040-3590.7.3.238

[ref15] Holt N. L. (2007). “Introduction: positive youth development through sport” in Positive youth development through sport (London: Routledge), 15–20.

[ref9005] Holt N. L. Neely K. C. Slater L. G. Camiré M. Côté J. Fraser-Thomas J.. (2017). A grounded theory of positive youth development through sport based on results from a qualitative meta-study. Int. Rev. Sport Exerc. Psychol. 10, 1–49. doi: 10.1080/1750984X.2016.1180704 27695511 PMC5020349

[ref16] Holt N. L. Tamminen K. A. Jones M. I. (2007). Promoting positive youth development through teaching games in physical education. Phys. Health Educ. J. 73.

[ref17] Ji X. Zheng S. Cheng C. Cheng L. Cronin L. (2022). Development and psychometric evaluation of the Chinese version of the life skills scale for physical education. Int. J. Environ. Res. Public Health 19:5324. doi: 10.3390/ijerph19095324 35564715 PMC9104646

[ref18] Kelloway E. K. (2014). Using Mplus for structural equation modeling: A researcher's guide. Thousand Oaks, California, USA: Sage Publications.

[ref19] Kline R. B. (1999). Book review: psychometric theory. J. Psychoeduc. Assess. 17, 275–280.

[ref20] Kline R. B. (2023). Principles and practice of structural equation modeling. New York, USA: Guilford Publications.

[ref21] Mardia K. V. (1970). Measures of multivariate skewness and kurtosis with applications. Biometrika 57, 519–530. doi: 10.1093/biomet/57.3.519

[ref23] Messick S. (1995). Validity of psychological assessment: validation of inferences from persons' responses and performances as scientific inquiry into score meaning. Am. Psychol. 50, 741–749. doi: 10.1037/0003-066X.50.9.741

[ref24] Mossman G. J. Robertson C. Williamson B. Cronin L. (2021). Development and initial validation of the life skills scale for sport–transfer scale (LSSS-TS). Psychol. Sport Exerc. 54:101906. doi: 10.1016/j.psychsport.2021.101906

[ref9006] Nunnally B. Bernstein I. (1994). Psychometric Theory. New York: Oxford University.

[ref9007] Pasquali L. (2009). Psychometrics. Revista da Escola de Enfermagem da USP. 43, 992–999. doi: 10.1590/S0080-62342009000500002

[ref25] Pierce S. Gould D. Camiré M. (2017). Definition and model of life skills transfer. Int. Rev. Sport Exerc. Psychol. 10, 186–211. doi: 10.1080/1750984X.2016.1199727

[ref26] Primo L. González-Hernández J. Yang Y. López de Subijana C. (2023). Predicting social skills in disadvantaged Chinese high school students through physical education. Front. Psychol. 14:1149223. doi: 10.3389/fpsyg.2023.1149223, PMID: 37179886 PMC10173878

[ref27] Ryan R. M. (2017). Self-determination theory: Basic psychological needs in motivation, development, and wellness. New York, USA: Guilford Press.

[ref28] Sousa V. D. Rojjanasrirat W. (2011). Translation, adaptation, and validation of instruments or scales for use in cross-cultural health care research: a clear and user-friendly guideline. J. Eval. Clin. Pract. 17, 268–274. doi: 10.1111/j.1365-2753.2010.01434.x 20874835

[ref30] Subasree R. Nair A. R. Ranjan R. (2014). The life skills assessment scale: the construction and validation of a new comprehensive scale for measuring life skills. J. Humanit. Soc. Sci. 19, 50–58. doi: 10.9790/0837-19195058, PMID: 38025460

[ref31] Tabachnick B. G. Fidell L. S. Ullman J. B. (2013). Using multivariate statistics, vol. 6. Boston, MA: Pearson, 497–516.

[ref33] Weinberg R. S. Gould D. (2023). Foundations of sport and exercise psychology. Champaign, Illinois, USA: Human Kinetics.

[ref34] World Health Organization. (2009). Preventing violence by developing life skills in children and adolescents. Geneva, Switzerland: World Health Organization.

[ref9008] Yuliang O. Qinqin Y. Caiyao Q. (2017). Development of a life skills evaluation scale for vocational college students from the perspective of experts. High. Educ. Exploration. 12, 98–102.

