# Supplementary material for: Cross-cultural validation and psychometric properties study of the Chinese college students’ life skills scale for sport—transfer scale
Source: Front Psychol. 2025 Jan 20;15:1431239. doi: 10.3389/fpsyg.2024.1431239 (PMC11788395; doi:10.3389/fpsyg.2024.1431239)
Supplement: Supplementary file 1 [file Table_1.docx]

Appendix：

***C-LSSS-TS***

Guidelines:

Life skills learned through sports by young people may transfer to other areas of life. The following questions pertain to the areas where you may transfer life skills. Please assess the extent to which you transfer life skills to each area for these questions.

All Questions: Please answer by circling the appropriate response to the right of each question. There are no right or wrong answers, so please answer as honestly as possible.

| 编号 | 项目 | 完全没有 | 有一点 | 一些 | 很多 | 非常多 |
| --- | --- | --- | --- | --- | --- | --- |
| 1 | 在社交环境中，我运用了体育活动培养的团队合作技能。 |  |  |  |  |  |
| 2 | 在家庭生活中，我运用了体育活动培养的团队合作技能。 |  |  |  |  |  |
| 3 | 在学校教育中，我运用了体育活动培养的团队合作能力。 |  |  |  |  |  |
| 4 | 在社区参与中，我运用了体育活动培养的团队合作技能。 |  |  |  |  |  |
| 5 | 在就业发展中，我运用了体育活动培养的团队合作技能。 |  |  |  |  |  |
| 6 | 在社交环境中，我运用了体育活动培养的目标设定技能。 |  |  |  |  |  |
| 7 | 在家庭生活中，我运用了体育活动培养的目标设定技能。 |  |  |  |  |  |
| 8 | 在学校教育中，我运用了体育活动培养的目标设定技能。 |  |  |  |  |  |
| 9 | 在社区参与中，我运用了体育活动培养的目标设定技能。 |  |  |  |  |  |
| 10 | 在就业发展中，我运用了体育活动培养的目标设定技能。 |  |  |  |  |  |
| 11 | 在社交环境中，我运用了体育活动培养的社交技能。 |  |  |  |  |  |
| 12 | 在家庭生活中，我运用了体育活动培养的社交技能。 |  |  |  |  |  |
| 13 | 在学校教育中，我运用了体育活动培养的社交技能。 |  |  |  |  |  |
| 14 | 在社区参与中，我运用了体育活动培养的社交技能。 |  |  |  |  |  |
| 15 | 在就业发展中，我运用了体育活动培养的社交技能。 |  |  |  |  |  |
| 16 | 在社交环境中，我运用了体育活动培养的问题解决与决策能力。 |  |  |  |  |  |
| 17 | 在家庭生活中，我运用了体育活动培养的问题解决与决策能力。 |  |  |  |  |  |
| 18 | 在学校教育中，我运用了体育活动培养的问题解决与决策能力。 |  |  |  |  |  |
| 19 | 在社区参与中，我运用了体育活动培养的问题解决与决策能力。 |  |  |  |  |  |
| 20 | 在就业发展中，我运用了体育活动培养的问题解决与决策能力。 |  |  |  |  |  |
| 21 | 在社交环境中，我运用了体育活动培养的情感技能。 |  |  |  |  |  |
| 22 | 在家庭生活中，我运用了体育活动培养的情感技能。 |  |  |  |  |  |
| 23 | 在学校教育中，我运用了体育活动培养的情感技能。 |  |  |  |  |  |
| 24 | 在社区参与中，我运用了体育活动培养的情感技能。 |  |  |  |  |  |
| 25 | 在就业发展中，我运用了体育活动培养的情感技能。 |  |  |  |  |  |
| 26 | 在社交环境中，我运用了体育活动培养的领导能力。 |  |  |  |  |  |
| 27 | 在家庭生活中，我运用了体育活动培养的领导能力。 |  |  |  |  |  |
| 28 | 在学校教育中，我运用了体育活动培养的领导能力。 |  |  |  |  |  |
| 29 | 在社区参与中，我运用了体育活动培养的领导能力。 |  |  |  |  |  |
| 30 | 在就业发展中，我运用了体育活动培养的领导能力。 |  |  |  |  |  |
| 31 | 在社交环境中，我运用了体育活动培养的时间管理技能。 |  |  |  |  |  |
| 32 | 在家庭生活中，我运用了体育活动培养的时间管理技能。 |  |  |  |  |  |
| 33 | 在学校教育中，我运用了体育活动培养的时间管理技能。 |  |  |  |  |  |
| 34 | 在社区参与中，我运用了体育活动培养的时间管理技能。 |  |  |  |  |  |
| 35 | 在就业发展中，我运用了体育活动培养的时间管理技能。 |  |  |  |  |  |
| 36 | 在社交环境中，我运用了体育活动培养的人际沟通技能。 |  |  |  |  |  |
| 37 | 在家庭生活中，我运用了体育活动培养的人际沟通技能。 |  |  |  |  |  |
| 38 | 在学校教育中，我运用了体育活动培养的人际沟通技能。 |  |  |  |  |  |
| 39 | 在社区参与中，我运用了体育活动培养的人际沟通技能。 |  |  |  |  |  |
| 40 | 在就业发展中，我运用了体育活动培养的人际沟通技能。 |  |  |  |  |  |

***The English translation of C-LSSS-TS***

| Number | Item | Not at all | A little | Some | A lot | Very much |
| --- | --- | --- | --- | --- | --- | --- |
| 1 | In social settings, I utilize teamwork skills cultivated through sports activities. |  |  |  |  |  |
| 2 | In family life, I utilize teamwork skills cultivated through sports activities. |  |  |  |  |  |
| 3 | In educational settings, I utilize teamwork skills cultivated through sports activities. |  |  |  |  |  |
| 4 | In community engagement, I utilize teamwork skills cultivated through sports activities. |  |  |  |  |  |
| 5 | In career development, I utilize teamwork skills cultivated through sports activities. |  |  |  |  |  |
| 6 | In social settings, I utilize goal-setting skills cultivated through sports activities. |  |  |  |  |  |
| 7 | In family life, I utilize goal-setting skills cultivated through sports activities. |  |  |  |  |  |
| 8 | In educational settings, I utilize goal-setting skills cultivated through sports activities. |  |  |  |  |  |
| 9 | In community engagement, I utilize goal-setting skills cultivated through sports activities. |  |  |  |  |  |
| 10 | In career development, I utilize goal-setting skills cultivated through sports activities. |  |  |  |  |  |
| 11 | In social settings, I utilize social skills cultivated through sports activities. |  |  |  |  |  |
| 12 | In family life, I utilize social skills cultivated through sports activities. |  |  |  |  |  |
| 13 | In educational settings, I utilize social skills cultivated through sports activities. |  |  |  |  |  |
| 14 | In community engagement, I utilize social skills cultivated through sports activities. |  |  |  |  |  |
| 15 | In career development, I utilize social skills cultivated through sports activities. |  |  |  |  |  |
| 16 | In social settings, I utilize problem-solving and decision-making abilities cultivated through sports activities. |  |  |  |  |  |
| 17 | In family life, I utilize problem-solving and decision-making abilities cultivated through sports activities. |  |  |  |  |  |
| 18 | In educational settings, I utilize problem-solving and decision-making abilities cultivated through sports activities. |  |  |  |  |  |
| 19 | In community engagement, I utilize problem-solving and decision-making abilities cultivated through sports activities. |  |  |  |  |  |
| 20 | In career development, I utilize problem-solving and decision-making abilities cultivated through sports activities. |  |  |  |  |  |
| 21 | In social settings, I utilize emotional skills cultivated through sports activities. |  |  |  |  |  |
| 22 | In family life, I utilize emotional skills cultivated through sports activities. |  |  |  |  |  |
| 23 | In educational settings, I utilize emotional skills cultivated through sports activities. |  |  |  |  |  |
| 24 | In community engagement, I utilize emotional skills cultivated through sports activities. |  |  |  |  |  |
| 25 | In career development, I utilize emotional skills cultivated through sports activities. |  |  |  |  |  |
| 26 | In social settings, I utilize leadership abilities cultivated through sports activities. |  |  |  |  |  |
| 27 | In family life, I utilize leadership abilities cultivated through sports activities. |  |  |  |  |  |
| 28 | In educational settings, I utilize leadership abilities cultivated through sports activities. |  |  |  |  |  |
| 29 | In community engagement, I utilize leadership abilities cultivated through sports activities. |  |  |  |  |  |
| 30 | In career development, I utilize leadership abilities cultivated through sports activities. |  |  |  |  |  |
| 31 | In social settings, I utilize time management skills cultivated through sports activities. |  |  |  |  |  |
| 32 | In family life, I utilize time management skills cultivated through sports activities. |  |  |  |  |  |
| 33 | In educational settings, I utilize time management skills cultivated through sports activities. |  |  |  |  |  |
| 34 | In community engagement, I utilize time management skills cultivated through sports activities. |  |  |  |  |  |
| 35 | In career development, I utilize time management skills cultivated through sports activities. |  |  |  |  |  |
| 36 | In social settings, I utilize interpersonal communication skills cultivated through sports activities. |  |  |  |  |  |
| 37 | In family life, I utilize interpersonal communication skills cultivated through sports activities. |  |  |  |  |  |
| 38 | In educational settings, I utilize interpersonal communication skills cultivated through sports activities. |  |  |  |  |  |
| 39 | In community engagement, I utilize interpersonal communication skills cultivated through sports activities. |  |  |  |  |  |
| 40 | In career development, I utilize interpersonal communication skills cultivated through sports activities. |  |  |  |  |  |
